# Concurrent chemoradiotherapy with S-1 *versus* platinum in the treatment of locoregionally advanced nasopharyngeal carcinoma: a multicenter, retrospective, propensity score-matched analysis

**DOI:** 10.3389/fphar.2024.1394754

**Published:** 2024-11-06

**Authors:** Chenbin Bian, Zhuangzhuang Zheng, Jing Su, Sitong Chang, Huiyuan Yu, Jindian Bao, Qin Zhao, Xin Jiang

**Affiliations:** ^1^ Department of Radiation Oncology and Jilin Provincial Key Laboratory of Radiation Oncology and Therapy, The First Hospital of Jilin University, Changchun, China; ^2^ NHC Key Laboratory of Radiobiology, School of Public Health of Jilin University, Changchun, China

**Keywords:** concurrent chemoradiotherapy, S-1, platinum, nasopharyngeal carcinoma, propensity score matching, prognosis, toxicity

## Abstract

**Objectives:**

Literature data are scarce on concurrent chemoradiotherapy (CCRT) with S-1 for locally advanced nasopharyngeal carcinoma (LANPC) treatment. This study compared the efficacy and safety of the S-1 *versus* platinum-based CCRT in LANPC treatment. Methods: This study enrolled 547 patients newly diagnosed with LANPC who underwent CCRT with S-1 or platinum at three institutions. Propensity score matching in a 1:1 ratio balancing baseline features was performed. Survival and adverse effects were compared between groups.

**Results:**

Of 160 patients in the cohort, 100 eligible were propensity score matched. Matched dataset analyses showed a higher 5-year overall survival rate (87.1% vs. 84.7%, *P* = 0.833), progression-free survival (79.6% vs. 75.5%, *P* = 0.669), locoregional recurrence-free survival (87.0% vs. 84.7%, *P* = 0.518), and distant metastasis-free survival (84.8% vs. 83.0%, *P* = 0.780) in the S-1 group than in the platinum-based CCRT group, although not statistically significant. Objective response rate (98.0% vs. 88.0%, *P* = 0.117) was significantly higher in the S-1 than in the platinum-based regimen, although it was not statistically reflected. Compared with platinum-based, those undergoing S-1-based chemotherapy demonstrated a higher incidence of grade 3 mucositis (20.0% vs. 2.0%, *P* = 0.016) in the S-1 group and a lower incidence of leukopenia (44.0% vs. 68.0%, *P* = 0.033), neutropenia (28.0% vs. 52.0%, *P* = 0.032), anemia (22.0% vs. 44.0%, *P* = 0.040), nephrotoxicity (4.0% vs. 20.0%, *P* = 0.028), and nausea/vomiting (30.0% vs. 56.0%, *P* = 0.019).

**Conclusion:**

The results suggest that S-1 can be used as a concurrent chemotherapy regimen during radiotherapy for patients with LANPC, since it presents a noninferior survival benefit compared with platinum and shows tolerable adverse effects.

## Introduction

Nasopharyngeal carcinoma (NPC) is a malignant epithelial tumor originating from the nasopharynx, with a unique and unbalanced endemic distribution and high incidence in East and Southeast Asia, especially in South China (Chua et al., 2016; Chen et al., 2019). Owing to the peculiar anatomic location of the nasopharynx, 70% of NPC patients present with an advanced stage at initial diagnosis, and locally advanced nasopharyngeal carcinoma (LANPC) has a high metastasis rate and relatively poor prognosis (You et al., 2017). In 2016, 52,000 new cases and 26,700 deaths occurred from NPC in China, caused by interactions between Epstein-Barr virus infection and genetic and environmental factors, such as alcohol consumption and smoking (Tang et al., 2016; Zheng et al., 2022). NPC is a distinct type of head and neck cancer regarding treatment response because of its sensitivity to radiotherapy and chemotherapy (Hu et al., 2017). Induction chemotherapy based on cisplatin, 5-florouracil, and docetaxel can improve the survival outcome of patients with LANPC; however, a limited long-term treatment regimen necessitates new strategies for further improvement (Liu et al., 2018).

Intensity-modulated radiotherapy (IMRT) is a recognized and effective method for the radical treatment of NPC, and the 10-year survival rate of early-stage patients (stage I-II) after radical radiotherapy can reach 90% (Bossi et al., 2021). The Intergroup 0,099 trial found that the survival endpoint of concurrent chemoradiotherapy (CCRT) and adjuvant chemotherapy was superior to that of radiotherapy alone, thus establishing CCRT as the standard treatment for LANPC (stages III-IVa) (Al-Sarraf et al., 1998). A meta-analysis of individual data from 19 randomized controlled trials showed that CCRT (with or without adjuvant chemotherapy) significantly improved overall survival (OS) compared with adjuvant chemotherapy or induction chemotherapy plus radiotherapy (Blanchard et al., 2015). Therefore, CCRT is the core treatment for LANPC. The National Comprehensive Cancer Network Guidelines (Caudell et al., 2022) recommend using a cisplatin 100 mg/m^2^ once every 3 weeks or 40 mg/m^2^ once a week in conjunction with radiotherapy (Caudell et al., 2022). Although patients with LANPC have a clear survival benefit from cisplatin-based CCRT, it is often associated with poor treatment adherence and quality of life due to the known adverse effects of cisplatin, including hematologic toxicity, gastrointestinal reactions, nephrotoxicity, ototoxicity, and neurotoxicity (Ben Ayed et al., 2020; Wu et al., 2021). Cisplatin-based concurrent chemoradiotherapy requires pre- and post-treatment fluid replacement during cisplatin administration to protect renal function, prolonging the duration of hospital stay (Tang et al., 2018). A prospective study showed no difference in efficacy between carboplatin-based CCRT and cisplatin-based regimens in LANPC treatment, with better carboplatin tolerability (Chitapanarux et al., 2007). However, these results were unconfirmed in two subsequent trials, which found no further improvement in OS in patients receiving carboplatin compared to those receiving radiotherapy alone or cisplatin-based CCRT, thus concluding that the carboplatin regimen is unsuitable for use with concurrent radiotherapy (Yau et al., 2006; Huang et al., 2012). Nedaplatin, a reported alternative to cisplatin-based concurrent chemoradiotherapy, had unignorable toxicity (Tang et al., 2018). Therefore, new chemotherapeutic agents with similar efficacy but less toxicity than platinum are urgently warranted.

S-1 is composed of tegafur (a precursor of 5-fluorouracil), gimeracil, and potassium oxonate with less toxicity as anti-tumor drugs; its ability to effectively inhibit DNA synthesis has also been shown to improve the efficacy of radiotherapy (Takiuchi and Ajani, 1998). The most well-known indication is the combination of cisplatin in the treatment of unresectable locally advanced or metastatic gastric cancer. S-1 monotherapy is effective and safe for gastric and lung cancer treatment (Kuyama et al., 2017). Moreover, S-1 concurrent radiotherapy improved survival in head and neck cancer by preventing distant metastasis and was equally effective and well tolerated in older patients with gastric, pancreatic, and non-small cell lung cancer (Ikeda et al., 2013; Kim et al., 2018; Sasaki et al., 2018). Recently, a prospective phase II study of LANPC showed that the 3-year progression-free survival (PFS), OS, locoregional recurrence-free survival (LRRFS), and distant metastasis-free survival (DMFS) rates after S-1 concurrent radiotherapy were 87.4%, 95.7%, 94.7%, and 91.5%, respectively, with mild toxicity (Lv et al., 2019). These results suggest that S-1 may be a new drug alternative to platinum. However, studies on S-1 concurrent radiotherapy in patients with LANPC are still limited. The clinical benefits and toxicity tolerance of the S-1 regimen has clinical benefits in compared with platinum-based concurrent radiotherapy require further exploration. Therefore, we performed a retrospective propensity score-matched analysis to compare the efficacy and safety of concurrent radiotherapy with S-1 and platinum in patients with LANPC.

## Patients and methods

### Patient selection

**FIGURE 1:**
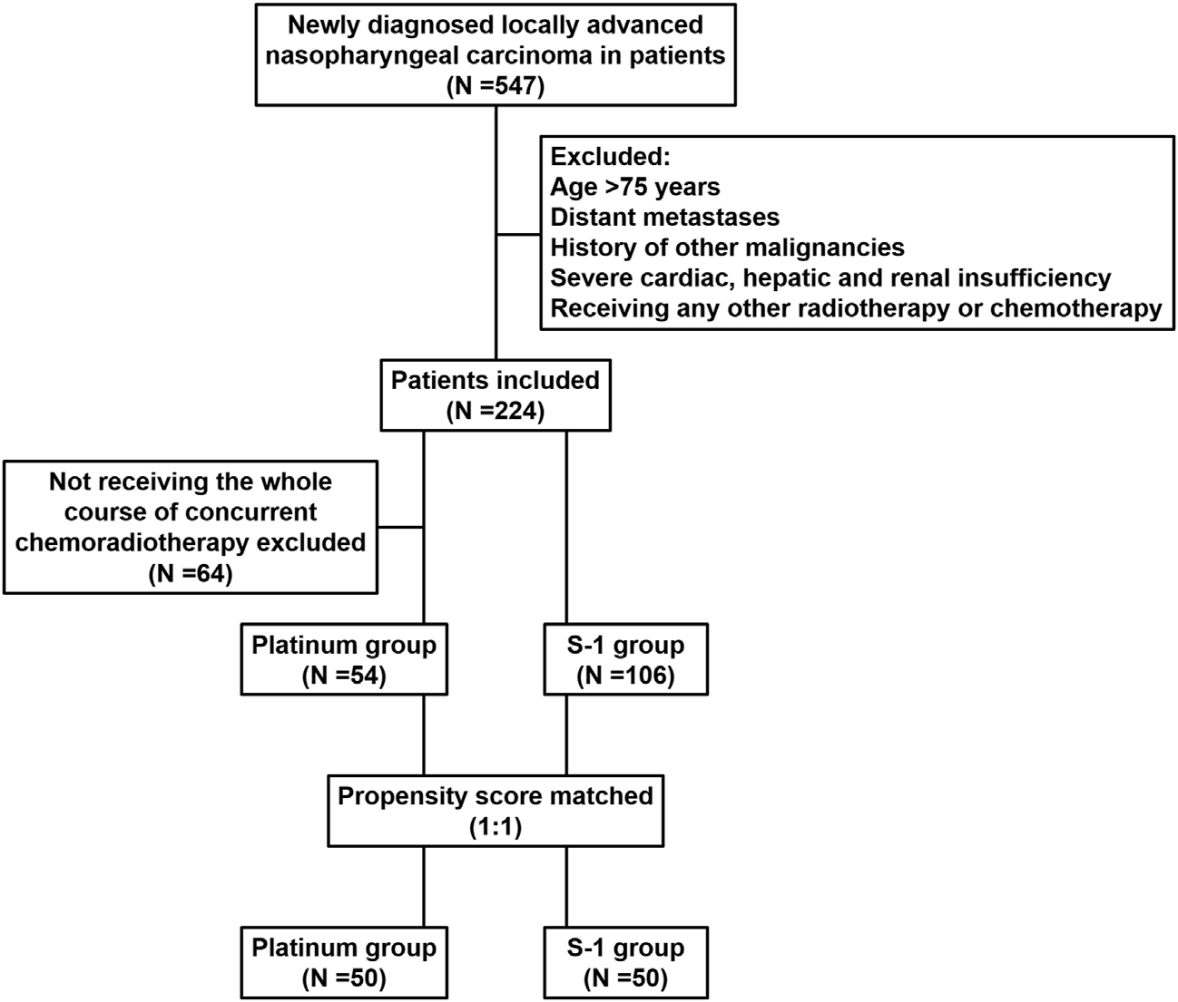
Retrospective study flow chart.

We reviewed the inpatient medical records of patients with LANPC treated with radiotherapy at the First Hospital of Jilin University, Sino-Japanese Friendship Hospital of Jilin University, and Jilin Cancer Hospital between January 2010 and December 2018. A total of 547 patients were identified, and the inclusion criteria were pathologically confirmed non-metastatic NPC according to the 8th edition of the Union for International Cancer Control/American Joint Committee on Cancer (UICC/AJCC) staging system; patients received radical IMRT combined with S-1 or platinum concurrent chemotherapy; Karnofsky Performance Status (KPS) ≥70. The exclusion criteria were age >75 years; previous malignancy or other concomitant malignant diseases; severe cardiac, hepatic, and renal insufficiency; and prior radiation therapy, chemotherapy, hormonal therapy, or molecular targeted therapy, less than 4 weeks after therapy completion and before initiating study medication (Figure 1). The patient’s pre-treatment assessment was as follows: a complete patient history, physical examination, routine blood test, biochemistry profiles, nasoendoscopy, computed tomography (CT), magnetic resonance imaging (MRI) of nasopharynx and neck, abdominal ultrasonography, whole-body bone scan or 18F-fluorodeoxyglucose positron emission tomography/computed tomography. The plasma Epstein-Barr virus (EBV) DNA titer was quantified before treatment using quantitative real-time polymerase chain reaction (qPCR) with a cut-off value of 500 copies/mL (Yu et al., 2021). This study was approved by the Ethics Committee of the First Hospital of Jilin University (protocol code: 2024-041).

### Radiotherapy

IMRT was administered to the nasopharynx and neck. The prescribed doses to the primary tumor region of the nasopharynx (GTVnx) were 68–76 Gy, enlarged cervical lymph nodes (GTVnd) were 66–70 Gy, high-risk clinical target volume (CTV1) were 60–64 Gy, and low-risk clinical target volume (CTV2) were 50–54 Gy. All patients were treated once a day, 5 times a week, for 30–33 fractions.

### Chemotherapy

The induction chemotherapy regimens included cisplatin plus 5-fluorouracil (PF; 80 mg/m^2^ d1 and 800 mg/m^2^ d1–d5, respectively), docetaxel plus cisplatin (TP; 75 mg/m^2^ d1 and 75 mg/m^2^ d1, respectively), and a triple combination of TPF (60 mg/m^2^ d1, 60 mg/m^2^ d1, and 600 mg/m^2^ d1–d5, respectively), repeated every 3 weeks for 2–3 cycles. A concurrent chemotherapy regimen was initiated on the first day of radiotherapy. Cisplatin or nedaplatin was administered via intravenous infusion at a dose of 100 mg/m^2^ every 3 weeks for a minimum of two cycles. S-1 60 mg was administered twice daily (orally after breakfast and dinner) for 2 consecutive weeks, subsequently stopped for 1 week, for a cycle of 3 weeks.

### Adverse events and follow-up

Treatment-related adverse events were graded according to the National Cancer Institute Common Toxicity Criteria version 5.0 (CTCAE V5.0). Tumor response was assessed 4 weeks after CCRT completion according to the Response Evaluation Criteria in Solid Tumors (RECIST 1.1). The follow-up period was calculated from the first day of treatment after LANPC diagnosis to the last follow-up or death date. Patients were examined every 3 months for the first 2 years, every 6 months for the next 3 years, and annually afterward. Nasopharyngoscopy, enhanced MRI of the head and neck, chest CT, and abdominal ultrasonography were routinely performed. The final follow-up period was December 2022.

### Statistical analysis

The study endpoints were PFS, OS, LRRFS, and DMFS. PFS was calculated from the treatment date to the occurrence of locoregional recurrence, distant metastasis, or death from any cause, whichever occurred first. OS was calculated from the treatment date to death from any cause or the last date known alive. LRRFS and DMFS were defined as the time from the first treatment day to the first locoregional recurrence and first distant metastasis, respectively.

Statistical analyses were performed using the SPSS 26.0 version. Survival analysis was performed using the Kaplan–Meier survival curve in GraphPad Prism 8.0.1. The chi-square test was used to compare the two treatment groups’ clinical characteristics, short-term efficacy, and adverse events. The Cox proportional hazards model was used for multivariate analysis. A 1:1 ratio was used for propensity score matching to create a cohort of patients receiving concurrent chemotherapy with platinum or S-1, with a caliper of 0.2 to control for potential bias. Matched covariates included age, sex, KPS, histological type, pre-treatment EBV DNA level, T stage, N stage, clinical stage, and induction chemotherapy. A value of *P* < 0.05 was considered statistically significant.

## Results

### Patient characteristics

Between January 2010 and December 2018, 224 of 547 patients diagnosed with LANPC by pathological examination met the inclusion criteria; 64 patients failed to complete three cycles of concurrent chemotherapy because of acute coronary syndrome, interstitial pneumonia, extremely severe liver and kidney injury, and poor compliance. Finally, of the 160 patients who met the propensity score matching criteria, 106 (66.3%) and 54 (33.7%) received S-1 and platinum, respectively. Before matching, patients who received platinum-based concurrent chemotherapy were more likely to receive induction chemotherapy (*P* < 0.001) and have a relatively late clinical-stage (*P* = 0.072). After propensity score matching analysis, 100 patients (50 pairs) were selected; the clinical characteristics of the S-1 and platinum groups were balanced, and no statistical difference was detected. Baseline characteristics of the study cohort are presented in Table 1.

**TABLE 1:** Baseline characteristics of patients with locally advanced nasopharyngeal carcinoma in the whole and propensity score matching datasets.

Characteristic	Observational dataset [cases (%)]				Propensity score-matched dataset [cases (%)]			
	Total	Platinum	S-1	P-value	Total	Platinum	S-1	P-value
Total	160	54	106		100	50	50	
Age				0.920				0.542
≤52 years	88 (55.0%)	30 (55.6%)	58 (54.7%)		59 (59.0%)	28 (56.0%)	31 (62.0%)	
>52 years	72 (45.0%)	24 (44.4%)	48 (45.3%)		41 (41.0%)	22 (44.0%)	19 (38.0%)	
Gender				0.562				0.362
Male	120 (75.0%)	42 (77.8%)	78 (73.6%)		74 (74.0%)	39 (78.0%)	35 (70.0%)	
Female	40 (25.0%)	12 (22.2%)	28 (26.4%)		26 (26.0%)	11 (22.0%)	15 (30.0%)	
Histology				1.000				1.000
I	7 (4.4%)	2 (3.7%)	5 (4.7%)		4 (4.0%)	2 (4.0%)	2 (4.0%)	
II–III	153 (95.6%)	52 (96.3%)	101 (95.3%)		96 (96.0%)	48 (96.0%)	48 (96.0%)	
T stage				0.208				1.000
T1	9 (5.6%)	2 (3.7%)	7 (6.6%)		5 (5.0%)	2 (4.0%)	3 (6.0%)	
T2	46 (28.7%)	11 (20.4%)	35 (33.0%)		19 (19.0%)	10 (20.0%)	9 (18.0%)	
T3	64 (40.0%)	23 (42.6%)	41 (38.7%)		46 (46.0%)	23 (46.0%)	23 (46.0%)	
T4	41 (25.6%)	18 (33.3%)	23 (21.7%)		30 (30.0%)	15 (30.0%)	15 (30.0%)	
N stage				0.106				0.438
N0	6 (3.8%)	1 (1.9%)	5 (4.7%)		6 (6.0%)	1 (2.0%)	5 (10.0%)	
N1	20 (12.5%)	8 (14.8%)	12 (11.3%)		13 (13.0%)	7 (14.0%)	6 (12.0%)	
N2	102 (63.7%)	29 (53.7%)	73 (68.9%)		57 (57.0%)	29 (58.0%)	28 (56.0%)	
N3	32 (20.0%)	16 (29.6%)	16 (15.1%)		24 (24.0%)	13 (26.0%)	11 (22.0%)	
Clinical stage				0.072				0.411
II	11 (6.9%)	4 (7.4%)	7 (6.6%)		10 (10.0%)	3 (6.0%)	7 (14.0%)	
III	85 (53.1%)	22 (40.7%)	63 (59.4%)		42 (42.0%)	22 (44.0%)	20 (40.0%)	
IV	64 (40.0%)	28 (51.9%)	36 (34.0%)		48 (48.0%)	25 (50.0%)	23 (46.0%)	
KPS				1.000				1.000
70–90	5 (3.1%)	2 (3.7%)	3 (2.8%)		4 (4.0%)	2 (4.0%)	2 (4.0%)	
≥90	155 (96.9%)	52 (96.3%)	103 (97.2%)		96 (96.0%)	48 (96.0%)	48 (96.0%)	
Pretreatment EBV DNA				1.000				1.000
<500 copies/mL	155 (93.8%)	51 (94.4%)	99 (93.4%)		96 (96.0%)	48 (96.0%)	48 (96.0%)	
≥500 copies/mL	10 (6.3%)	3 (5.6%)	7 (6.6%)		4 (4.0%)	2 (4.0%)	2 (4.0%)	
Chemotherapy				0.001				0.836
Concurrent	89 (55,6%)	20 (37.0%)	69 (65.1%)		41 (41.0%)	20 (40.0%)	21 (42.0%)	
Induction + concurrent	71 (44.4%)	34 (63.0%)	37 (34.9%)		59 (59.0%)	30 (60.0%)	29 (58.0%)	

### Survival outcomes

**FIGURE 2:**
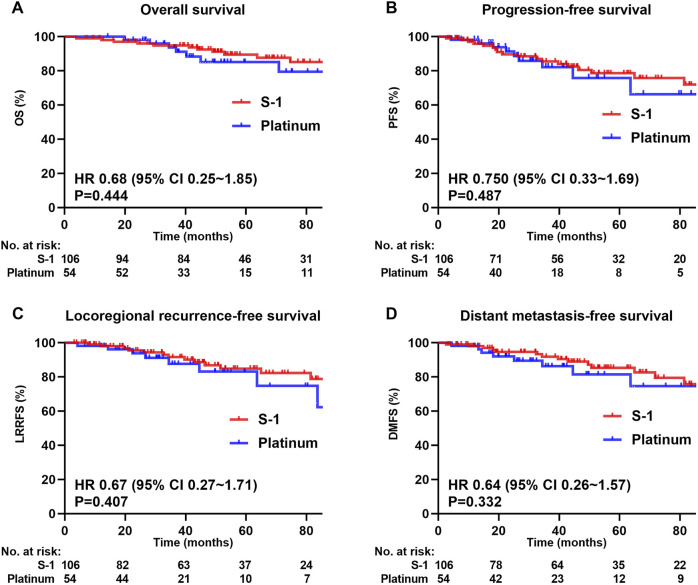
Kaplan-Meier survival curves of 160 patients in the whole cohort. **(A)** Overall survival, **(B)** Progression-free survival, **(C)** Locoregional recurrence-free survival, **(D)** Distant metastasis-free survival. We calculated p values using the unadjusted log-rank test and HRs and their associated 95% CIs using a univariate cox regression analysis.

The median follow-up for the whole cohort was 51.8 months (range 3.9–148.4 months), wherein 18 patients (11.3%) died, 22 (13.8%) had local recurrence, and 23 (14.4%) had distant metastases. Figure 2 shows survival curves for the entire cohort. The 5-year OS, PFS, LRRFS, and DMFS rates in the S-1 group and platinum group were 89.5% and 85.1% (*P* = 0.444; Figure 2A), 78.6% and 75.8% (*P* = 0.487; Figure 2B), 84.9% and 83.1% (*P* = 0.407; Figure 2C), and 85.4% and 81.5% (*P* = 0.332; Figure 2D), respectively.

Multivariate analysis was performed using the Cox proportional hazards model to adjust for various prognostic factors (Table 2). Consistent with the univariate results, multivariate analysis showed that 5-year OS (HR = 0.66, 95% CI = 0.23–1.87; *P* = 0.434), PFS (HR = 1.05, 95% CI = 0.45–2.42; *P* = 0.916), LRRFS (HR = 1.12, 95% CI = 0.43–2.89; *P* = 0.814), and DMFS (HR = 0.88, 95% CI = 0.35–2.24; *P* = 0.790) remained similar in both treatment groups.

**TABLE 2:** Multivariate analysis of prognostic factors for the whole cohort.

Variable	PFS			LRRFS			DMFS			OS		
	B	HR (95%CI)	P-value	B	HR (95%CI)	P-value	B	HR (95%CI)	P-value	B	HR (95%CI)	P-value
Age												
≤52 years	0			0			0			0		
>52 years	0.16	1.18 (0.56–2.47)	0.671	0.24	1.27 (0.54–2.95)	0.586	0.07	1.08 (0.46–2.53)	0.814	−0.07	0.93 (0.36–2.41)	0.880
Gender												
Female	0			0			0			0		
Male	0.79	2.20 (0.80–6.05)	0.127	0.98	2.68 (0.74–9.61)	0.132	0.87	2.38 (0.76–7.43)	0.165	1.32	3.73 (0.81–17.15)	0.091
T stage												
T1–2	0			0			0			0		
T3–4	0.53	1.69 (0.71–4.02)	0.233	0.97	2.64 (0.85–8.22)	0.095	−0.19	0.83 (0.33–2.08)	0.688	0.64	1.90 (0.64–5.70)	0.250
N stage												
N0–1	0			0			0			0		
N2–3	−0.17	0.84 (0.31–2.29)	0.737	−0.13	0.88 (0.25–3.07)	0.839	−0.07	0.93 (0.30–2.91)	0.820	−0.69	0.50 (0.16–1.60)	0.243
Clinical stage												
II–III	0			0			0			0		
IV	−0.12	0.89 (0.39–2.03)	0.778	0.18	1.20 (0.48–2.99)	0.692	−0.16	0.86 (0.34–2.19)	0.918	−0.59	0.56 (0.19–1.68)	0.298
Chemotherapy												
Concurrent	0			0			0			0		
Induction + concurrent	0.64	1.90 (0.84–4.33)	0.125	0.47	1.60 (0.63–4.04)	0.323	0.52	1.68 (0.67–4.11)	0.229	−0.17	0.85 (0.29–2.44)	0.759
Concurrent chemotherapy												
Platinum	0			0			0			0		
S-1	0.05	1.05 (0.45–2.42)	0.916	0.11	1.12 (0.43–2.89)	0.814	−0.13	0.88 (0.35–2.24)	0.790	−0.42	0.66 (0.23–1.87)	0.434

### Propensity score

**FIGURE 3:**
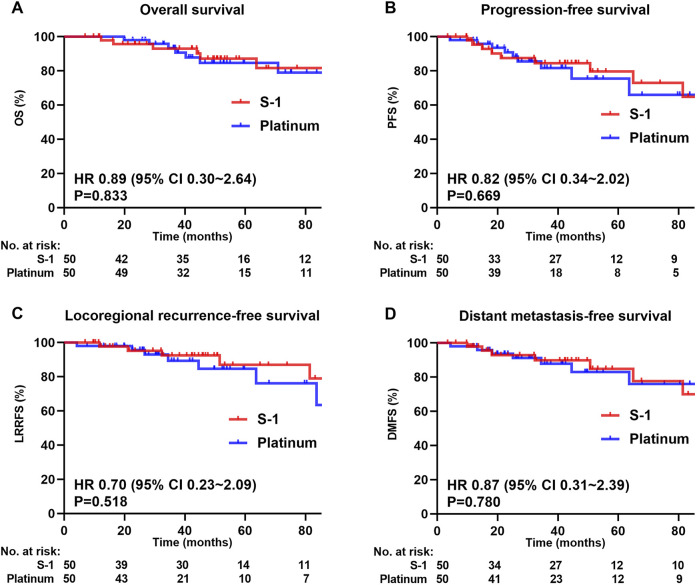
Kaplan-Meier survival curves of 100 patients in the matched cohort. **(A)** Overall survival, **(B)** Progression-free survival, **(C)** Locoregional recurrence-free survival, **(D)** Distant metastasis-free survival. We calculated p values using the unadjusted log-rank test and HRs and their associated 95% CIs using a univariate Cox regression analysis.

The median follow-up for the matched cohort was 49.7 months (range 6.9–148.4 months). In the matched cohort, 6 (12.0%) patients died, 6 (12.0%) developed local recurrence, and 7 (14.0%) had distant metastasis in the S-1 group; 7 (14.0%) and 8 (16.0%) dead patients had developed local recurrence and distant metastasis, respectively, in the platinum group. The S-1 group achieved a higher absolute 5-year OS (87.1% vs 84.7%, *P* = 0.833; Figure 3A), PFS (79.6% vs. 75.5%, *P* = 0.669; Figure 3B), LRRFS (87.0% vs 84.7%, *P* = 0.518; Figure 3C), and DMFS (84.8% vs 83.0%, *P* = 0.780; Figure 3D) than the platinum group, although the difference was not statistically significant.

In multivariate analysis (Table 3), the difference in the improvement of OS (HR = 0.91, 95% CI = 0.29–2.87; *P* = 0.876), PFS (HR = 0.93, 95% CI = 0.36–2.39; *P* = 0.883), LRRFS (HR = 0.85, 95% CI = 0.26–2.81; *P* = 0.789), and DMFS (HR = 0.91, 95% CI = 0.31–2.73; *P* = 0.871) between S-1 and platinum regimens was not statistically significant.

**TABLE 3:** Adjusted cox multivariate analyses of prognostic factors for the matched cohort.

Variable	PFS			LRRFS			DMFS			OS		
	B	HR (95%CI)	P-value	B	HR (95%CI)	P-value	B	HR (95%CI)	P-value	B	HR (95%CI)	P-value
Age												
≤52 years	0			0			0			0		
>52 years	0.02	1.02 (0.40–2.60)	0.964	−0.16	0.85 (0.26–2.78)	0.789	0.19	1.21 (0.41–3.64)	0.729	0.04	1.04 (0.34–3.23)	0.945
Gender												
Female	0			0			0			0		
Male	0.34	1.41 (0.47–4.20)	0.541	0.24	1.27 (0.29–5.65)	0.750	0.26	1.29 (0.37–4.58)	0.690	1.04	2.82 (0.58–13.75)	0.200
T stage												
T1–2	0			0			0			0		
T3–4	0.87	2.39 (0.61–9.35)	0.210	0.61	1.84 (0.68–6.16)	0.202	−0.45	0.64 (0.18–2.20)	0.475	0.97	2.63 (0.47–14.82)	0.274
N stage												
N0–1	0			0			0			0		
N2–3	−0.36	0.70 (0.19–2.64)	0.597	−0.33	0.72 (0.08–6.17)	0.761	0.43	1.54 (0.35–6.84)	0.571	−0.74	0.48 (0.11–2.09)	0.327
Clinical stage												
II–III	0			0			0			0		
IV	−0.52	0.60 (0.22–1.61)	0.307	−0.32	0.73 (0.23–2.30)	0.692	−0.82	0.44 (0.13–1.46)	0.181	−0.41	0.66 (0.21–2.11)	0.487
Chemotherapy												
Concurrent	0			0			0			0		
Induction + concurrent	0.47	1.60 (0.60–4.24)	0.345	0.06	1.06 (0.35–3.55)	0.924	0.55	1.73 (0.71–4.37)	0.298	0.02	1.02 (0.33–3.17)	0.977
Concurrent chemotherapy												
Platinum	0			0			0			0		
S-1	−0.07	0.93 (0.36–2.39)	0.883	−0.16	0.85 (0.26–2.81)	0.789	−0.09	0.91 (0.31–2.73)	0.871	−0.09	0.91 (0.29–2.87)	0.876

Subsequently, we evaluated the short-term efficacy of the two treatment groups based on tumor response 4 weeks after the CCRT (Table 4). The disease control rate (DCR) was similar in the S-1 and platinum groups; however, the objective response rate (ORR) was significantly higher in the S-1 group than in the platinum group (98.0% vs 88.0%, *P* = 0.117), although it was not statistically reflected.

**TABLE 4:** Analysis of tumor response in the matched dataset.

Characteristic	Total [cases (%)]	Platinum [cases (%)]	S-1 [cases (%)]	P-value
Tumor response				0.117
SD	7 (7.0%)	6 (12.0%)	1 (2.0%)	
PR	28 (28.0%)	11 (22.0%)	17 (34.0%)	
CR	65 (65%)	33 (66.0%)	32 (64.0%)	
ORR	93 (93.0%)	44 (88.0%)	49 (98.0%)	
DCR	100 (100%)	50 (100%)	50 (100%)	

### Adverse events

Table 5 summarizes the adverse events associated with chemoradiotherapy in matched cohorts. Concerning hematologic toxicity, the incidences of leukopenia (68.0% vs 44.0%, *P* = 0.033), neutropenia (52.0% vs. 28.0%, *P* = 0.032), and anemia (44.0% vs. 22.0%, *P* = 0.040) were higher in the platinum group than in the S-1 group. In addition, one patient in the platinum group had grade 4 leukopenia, and two patients had agranulocytosis. In the platinum group, nephrotoxicity (20.0% vs. 4.0%, *P* = 0.028) and nausea or vomiting (56.0% vs 30.0%, *P* = 0.019) were more common; however, the incidence of grade 3 oral mucositis (20.0% vs. 2.0%, *P* = 0.016) was significantly higher in the S-1 group than that in the platinum group.

**TABLE 5:** Chemoradiotherapy-related adverse events in the matched cohort.

Adverse events	Platinum (cases [%])	S-1 (cases [%])	P-value
Leukopenia			0.033
G0	16 (32.0%)	28 (56.0%)	
G1–2	24 (48.0%)	19 (38.0%)	
G3	9 (18.0%)	3 (6.0%)	
G4	1 (2.0%)	0	
Neutropenia			0.032
G0	24 (48.0%)	36 (72.0%)	
G1–2	20 (40.0%)	12 (24.0%)	
G3	4 (8.0%)	2 (4.0%)	
G4	2 (4.0%)	0	
Anemia			0.040
G0	28 (56.0%)	39 (78.0%)	
G1–2	21 (42.0%)	10 (20.0%)	
G3	1 (2.0%)	1 (2.0%)	
Thrombocytopenia			0.125
G0	37 (74.0%)	45 (90.0%)	
G1–2	10 (20.0%)	4 (8.0%)	
G3	3 (6.0%)	1 (2.0%)	
Mucositis			0.016
G0	8 (16.0%)	6 (12.0%)	
G1–2	41 (82.0%)	34 (68.0%)	
G3	1 (2.0%)	10 (20.0%)	
Radiodermatitis			0.660
G0	13 (26.0%)	15 (30.0%)	
G1–2	37 (74.0%)	34 (68.0%)	
G3	0	1 (2.0%)	
Nausea/vomiting			0.019
G0	22 (44.0%)	35 (70.0%)	
G1–2	24 (48.0%)	14 (28.0%)	
G3	4 (8.0%)	1 (2.0%)	
Xerostomia			0.086
G0	7 (14.0%)	14 (28.0%)	
G1–2	43 (86.0%)	36 (72.0%)	
G3	0	0	
Nephrotoxicity			0.028
G0	40 (80.0%)	48 (96.0%)	
G1–2	9 (18.0%)	2 (4.0%)	
G3	1 (2.0%)	0	
Hepatotoxicity			0.338
G0	43 (86.0%)	46 (92.0%)	
G1–2	7 (14.0%)	4 (8.0%)	
G3	0	0	
Ototoxicity			1.000
G0	48 (96.0%)	49 (98.0%)	
G1–2	2 (4.0%)	1 (2.0%)	
G3	0	0	

## Discussion

For patients with LANPC, CCRT is considered the standard treatment option, and its survival benefit with or without adjuvant chemotherapy is far greater than that of radiotherapy alone (Zhang et al., 2005; Lee et al., 2010). Several randomized trials have demonstrated the role of cisplatin-based CCRT in treating LANPC (Al-Sarraf et al., 2023). However, the comparable efficacy of S-1 as an oral chemotherapeutic agent remains unclear. Currently, only one prospective single-arm phase II study has evaluated the intervention effects of S-1 combined with IMRT for LANPC (Lv et al., 2019). Previous evidence suggests that randomized controlled trials primarily examine pharmaceutical intervention efficacy in controlled settings, whereas estimating true clinical effectiveness requires support from real-world evidence and network meta-analyses (Ford and Norrie, 2016). In the present study, we used propensity score matching analysis to eliminate the influence of confounding factors while assessing the curative effect and toxicity of CCRT with S-1 and platinum every 3 weeks for LANPC in the IMRT. To our knowledge, this is the first study to directly compare and evaluate the efficacy of S-1 and platinum in LANPC using real-world data, thus providing valuable insights into these treatment strategies in clinical practice.

Our retrospective study results showed that S-1 concurrent radiotherapy had comparable 5-year OS, PFS, LRRFS, and DMFS rates to platinum in whole and matched cohorts. These expected results have several plausible explanations. First, modern radiation techniques and equipment can improve treatment outcomes by delivering high radiation doses directly to target tissues (Beaton et al., 2019). A prospective study reported that IMRT has better local tumor control (91.7% vs 84.0%, *P* = 0.049) and survival (79.6% vs 67.1%, *P* = 0.001) rates than two-dimensional conventional radiation therapy (2D-CRT), especially in patients with LANPC (Peng et al., 2012). In addition, induction chemotherapy plus concurrent chemoradiotherapy has also been shown to be associated with favorable 5-year OS (85.6% vs 77.7%, *P* = 0.042), LRRFS (90.7% vs. 83.8%, *P* = 0.044), and DMFS (88.0% vs 79.8%, *P* = 0.030) rates (Li et al., 2019). Although more patients in the platinum-based group received induction chemotherapy (63.0% vs 34.9%, *P* = 0.001) than in the entire cohort, the survival benefit remained unchanged, possibly because of the greater proportion of stage IV patients (51.9% vs 34.0%, *P* = 0.072) in the platinum-based group. In our study, 5-year OS (HR = 0.91, 95% CI = 0.29–2.87; *P* = 0.876), PFS (HR = 0.93, 95% CI = 0.36–2.39; *P* = 0.883), LRRFS (HR = 0.85, 95% CI = 0.26–2.81; *P* = 0.789), and DMFS (HR = 0.91, 95% CI = 0.31–2.73; *P* = 0.871) were higher in the S-1 group than in the platinum, although this difference was not statistically significant. S-1 may improve short-term efficacy (ORR: 98.0% vs. 88.0%, *P* = 0.117), mainly owing to its radiosensitization or synergistic effect with radiotherapy, affecting long-term survival. This result contradicts common sense, which we considered possible because 4 weeks after treatment was not long enough for some patients to see significant change, and tumor shrinkage could continue for the first few weeks after completion of treatment.

Based on these findings, the therapeutic effects of the S-1 regimen should not be underestimated. A prospective study of 105 patients exploring the antitumor effect of 2D-CRT combined with S-1 in LANPC found that 2-year OS and PFS rates were 86.2% and 81.3%, respectively (Wen et al., 2015). Another study mentioned above showed that S-1 plus IMRT resulted in a 3-year OS of 95.7% and PFS of 87.4% (Lv et al., 2019). The marked difference in PFS and OS between these studies and the present study was chiefly attributed to the short follow-up period. Significant differences appear gradually; patients with NPC require more than 3 years of follow-up to determine the treatment effect, especially after 5 years (Liu et al., 2018). Another difference that must be addressed is drug toxicity. In the matched cohort of this study, 20% of the patients in the S-1 group developed grade 3 mucositis, which was higher than that reported in two previous studies. The main reason may be the serum 5-fluorouracil concentration increase due to a high dose of S-1. Therefore, further studies are needed to determine the optimal use of S-1 to balance toxicity and efficacy in the precision radiotherapy era.

Regarding adverse effects, the S-1-based CCRT regimen was well tolerated in this study compared to the platinum regimen. Many patients experience platinum-related toxicities and have relatively poor compliance. Tang et al. showed that grade 3 leukopenia, neutropenia, and nephrotoxicity were 22%, 12%, and 11% in the cisplatin group and 23%, 12%, and 4% in the nedaplatin group, respectively, similar to the results of our study (Tang et al., 2018). Leukopenia (6%), neutropenia (4%), and nephrotoxicity (0%) were uncommon in the S-1 group. Therefore, nearly all patients completed the planned treatment. Although mucositis was the most common restrictive factor for CCRT with S-1, this adverse effect was easily managed using growth factors and antibiotics (Elad et al., 2020). In recent years, docetaxel combined with radiotherapy is reportedly safe and effective for LANPC; however, the incidence of grade 3 mucositis is much higher than that with S-1 (74.5% vs 20.0%), limiting the widespread use of docetaxel-based CCRT in patients with LANPC (Liao et al., 2019). It has been reported that the anti-epidermal growth factor receptor (EGFR) monoclonal antibodies cetuximab and nimotuzumab combined with IMRT can maximize the survival rate of patients with LANPC, with a 3-year OS rate of 91.7% and a severe hematologic toxicity rate of only 3.4%–4.7% (You et al., 2017). Another large-data retrospective study showed that patients with stage III-IVB NPC who received IMRT plus nimotuzumab had a significantly higher 5-year OS rate than those who received IMRT alone (89.9% vs. 78.3%, *P* = 0.006) (Zhi-Qiang et al., 2019). However, cetuximab or nimotuzumab are too expensive to treat for most NPC patients in China, which limits the widespread use of concurrent chemoradiotherapy based on anti-EGFR antibodies in patients with LANPC. Additionally, oral administration is more convenient than intravenous infusion. Patients can take S-1 orally anytime and anywhere to complete the current treatment, saving the time for traveling to the hospital during the day to receive platinum infusions, which also substantially improves the compliance of outpatients and day ward patients. Therefore, S-1 combined with IMRT is safe and convenient for patients with platinum-intolerant LANPC, especially older patients and those who experience severe platinum-related adverse reactions during induction chemotherapy.

Since this retrospective study was based on real-world data, it had certain limitations. First, different clinicians use different treatment strategies for the same disease, and a certain degree of understandable deviation exists. Second, part of the collection of adverse events relies entirely on patient narratives, which may lead to the misgrading of toxic reactions. Although we eliminated selection bias using propensity score matching, such as age, sex, KPS, pre-treatment EBV DNA level, T stage, N stage, and clinical stage, it is unknown whether residual confounding factors remain. Further prospective studies with larger sample sizes are warranted.

## Conclusion

CCRT based on S-1 regimen showed a non-inferior survival benefit compared with platinum in LANPC, and the toxicity was tolerable. The spectrum of adverse reactions was different between the two drugs, with platinum having a higher incidence of hematological toxicity, gastrointestinal reactions and nephrotoxicity, while S-1 showed significant mucositis.

## Data Availability

The original contributions presented in the study are included in the article/supplementary material, further inquiries can be directed to the corresponding authors.

## Abbreviations

CCRT, concurrent chemoradiotherapy; CT, computed tomography; LANPC, locally advanced nasopharyngeal carcinoma; NPC, nasopharyngeal carcinoma; IMRT, Intensity-modulated radiotherapy; 2D-CRT, two-dimensional conventional radiation therapy; OS, overall survival; PFS, progression-free survival; LRRFS, locoregional recurrence-free survival; DMFS, distant metastasis-free survival; MRI, magnetic resonance imaging; EBV, Epstein-Barr virus; EGFR, epidermal growth factor receptor; qPCR, quantitative real-time polymerase chain reaction; DCR, disease control rate; ORR, objective response rate.

## References

[B1] Al-Sarraf M. LeBlanc M. Giri P. G. Fu K. K. Cooper J. Vuong T. (1998). Chemoradiotherapy versus radiotherapy in patients with advanced nasopharyngeal cancer: phase III randomized Intergroup study 0099. J. Clin. Oncol. 16 (4), 1310–1317. 10.1200/jco.1998.16.4.1310 9552031

[B2] Al-Sarraf M. LeBlanc M. Giri P. G. Fu K. K. Cooper J. Vuong T. (2023). Chemoradiotherapy versus radiotherapy in patients with advanced nasopharyngeal cancer: phase III randomized Intergroup study 0099. J. Clin. Oncol. 41 (24), 3965–3972. 10.1200/jco.22.02764 37586209

[B3] Beaton L. Bandula S. Gaze M. N. Sharma R. A. (2019). How rapid advances in imaging are defining the future of precision radiation oncology. Br. J. Cancer 120 (8), 779–790. 10.1038/s41416-019-0412-y 30911090 PMC6474267

[B4] Ben Ayed W. Ben Said A. Hamdi A. Mokrani A. Masmoudi Y. Toukabri I. (2020). Toxicity, risk factors and management of cisplatin-induced toxicity: a prospective study. J. Oncol. Pharm. Pract. 26 (7), 1621–1629. 10.1177/1078155219901305 32046580

[B5] Blanchard P. Lee A. Marguet S. Leclercq J. Ng W. T. Ma J. (2015). Chemotherapy and radiotherapy in nasopharyngeal carcinoma: an update of the MAC-NPC meta-analysis. Lancet Oncol. 16 (6), 645–655. 10.1016/s1470-2045(15)70126-9 25957714

[B6] Bossi P. Chan A. T. Licitra L. Trama A. Orlandi E. Hui E. P. (2021). Nasopharyngeal carcinoma: ESMO-EURACAN Clinical Practice Guidelines for diagnosis, treatment and follow-up(†). Ann. Oncol. 32 (4), 452–465. 10.1016/j.annonc.2020.12.007 33358989

[B7] Caudell J. J. Gillison M. L. Maghami E. Spencer S. Pfister D. G. Adkins D. (2022). NCCN Guidelines® insights: head and neck cancers, version 1.2022. J. Natl. Compr. Canc Netw. 20 (3), 224–234. 10.6004/jnccn.2022.0016 35276673

[B8] Chen Y. P. Chan A. T. C. Le Q. T. Blanchard P. Sun Y. Ma J. (2019). Nasopharyngeal carcinoma. Lancet 394 (10192), 64–80. 10.1016/s0140-6736(19)30956-0 31178151

[B9] Chitapanarux I. Lorvidhaya V. Kamnerdsupaphon P. Sumitsawan Y. Tharavichitkul E. Sukthomya V. (2007). Chemoradiation comparing cisplatin versus carboplatin in locally advanced nasopharyngeal cancer: randomised, non-inferiority, open trial. Eur. J. Cancer 43 (9), 1399–1406. 10.1016/j.ejca.2007.03.022 17467265

[B10] Chua M. L. K. Wee J. T. S. Hui E. P. Chan A. T. C. (2016). Nasopharyngeal carcinoma. Lancet 387 (10022), 1012–1024. 10.1016/s0140-6736(15)00055-0 26321262

[B11] Elad S. Cheng K. K. F. Lalla R. V. Yarom N. Hong C. Logan R. M. (2020). MASCC/ISOO clinical practice guidelines for the management of mucositis secondary to cancer therapy. Cancer 126 (19), 4423–4431. 10.1002/cncr.33100 32786044 PMC7540329

[B12] Ford I. Norrie J. (2016). Pragmatic trials. N. Engl. J. Med. 375 (5), 454–463. 10.1056/NEJMra1510059 27518663

[B13] Hu J. Kong L. Gao J. Hu W. Guan X. Lu J. J. (2017). Use of radiation therapy in metastatic nasopharyngeal cancer improves survival: a seer analysis. Sci. Rep. 7 (1), 721. 10.1038/s41598-017-00655-1 28389658 PMC5428776

[B14] Huang P. Y. Cao K. J. Guo X. Mo H. Y. Guo L. Xiang Y. Q. (2012). A randomized trial of induction chemotherapy plus concurrent chemoradiotherapy versus induction chemotherapy plus radiotherapy for locoregionally advanced nasopharyngeal carcinoma. Oral Oncol. 48 (10), 1038–1044. 10.1016/j.oraloncology.2012.04.006 22591726

[B15] Ikeda M. Ioka T. Ito Y. Yonemoto N. Nagase M. Yamao K. (2013). A multicenter phase II trial of S-1 with concurrent radiation therapy for locally advanced pancreatic cancer. Int. J. Radiat. Oncol. Biol. Phys. 85 (1), 163–169. 10.1016/j.ijrobp.2012.03.059 22677367

[B16] Kim M. J. Kong S. Y. Nam B. H. Kim S. Park Y. I. Park S. R. (2018). A randomized phase II study of S-1 versus capecitabine as first-line chemotherapy in elderly metastatic gastric cancer patients with or without poor performance status: clinical and pharmacogenetic results. Pharmacogenet Genomics 28 (1), 23–30. 10.1097/fpc.0000000000000320 29189588

[B17] Kuyama S. Ochi N. Bessho A. Hotta K. Ikeda G. Kishino D. (2017). A phase II trial of carboplatin plus S-1 for elderly patients with advanced non-small-cell lung cancer with wild-type epidermal growth factor receptor: the Okayama Lung Cancer Study Group Trial 1202. Lung Cancer 112, 188–194. 10.1016/j.lungcan.2017.08.010 29191594

[B18] Lee A. W. Tung S. Y. Chua D. T. Ngan R. K. Chappell R. Tung R. (2010). Randomized trial of radiotherapy plus concurrent-adjuvant chemotherapy vs radiotherapy alone for regionally advanced nasopharyngeal carcinoma. J. Natl. Cancer Inst. 102 (15), 1188–1198. 10.1093/jnci/djq258 20634482

[B19] Li W. F. Chen N. Y. Zhang N. Hu G. Q. Xie F. Y. Sun Y. (2019). Concurrent chemoradiotherapy with/without induction chemotherapy in locoregionally advanced nasopharyngeal carcinoma: long-term results of phase 3 randomized controlled trial. Int. J. Cancer 145 (1), 295–305. 10.1002/ijc.32099 30613964

[B20] Liao J. F. Zhang Q. Du X. J. Lan M. Liu S. Xia Y. F. (2019). Concurrent chemoradiotherapy with weekly docetaxel versus cisplatin in the treatment of locoregionally advanced nasopharyngeal carcinoma: a propensity score-matched analysis. Cancer Commun. (Lond) 39 (1), 40. 10.1186/s40880-019-0380-x 31248459 PMC6598276

[B21] Liu G. Y. Lv X. Wu Y. S. Mao M. J. Ye Y. F. Yu Y. H. (2018). Effect of induction chemotherapy with cisplatin, fluorouracil, with or without taxane on locoregionally advanced nasopharyngeal carcinoma: a retrospective, propensity score-matched analysis. Cancer Commun. (Lond) 38 (1), 21. 10.1186/s40880-018-0283-2 29764487 PMC5993041

[B22] Lv T. Wang Y. Ou D. Liu P. Qin S. Liu L. (2019). IMRT combined with S-1 concurrent chemoradiotherapy in locally advanced nasopharyngeal carcinoma: a prospective phase II study. Invest. New Drugs 37 (2), 352–359. 10.1007/s10637-018-00720-0 30617703

[B23] Peng G. Wang T. Yang K. Y. Zhang S. Zhang T. Li Q. (2012). A prospective, randomized study comparing outcomes and toxicities of intensity-modulated radiotherapy vs. conventional two-dimensional radiotherapy for the treatment of nasopharyngeal carcinoma. Radiother. Oncol. 104 (3), 286–293. 10.1016/j.radonc.2012.08.013 22995588

[B24] Sasaki Y. Iwasa S. Okazaki S. Goto M. Kojima Y. Naganuma A. (2018). A phase II study of combination therapy with oral S-1 and cisplatin in elderly patients with advanced gastric cancer. Gastric Cancer 21 (3), 439–445. 10.1007/s10120-017-0753-2 28766263

[B25] Takiuchi H. Ajani J. A. (1998). Uracil-tegafur in gastric carcinoma: a comprehensive review. J. Clin. Oncol. 16 (8), 2877–2885. 10.1200/jco.1998.16.8.2877 9704742

[B26] Tang L. L. Chen W. Q. Xue W. Q. He Y. Q. Zheng R. S. Zeng Y. X. (2016). Global trends in incidence and mortality of nasopharyngeal carcinoma. Cancer Lett. 374 (1), 22–30. 10.1016/j.canlet.2016.01.040 26828135

[B27] Tang L. Q. Chen D. P. Guo L. Mo H. Y. Huang Y. Guo S. S. (2018). Concurrent chemoradiotherapy with nedaplatin versus cisplatin in stage II-IVB nasopharyngeal carcinoma: an open-label, non-inferiority, randomised phase 3 trial. Lancet Oncol. 19 (4), 461–473. 10.1016/s1470-2045(18)30104-9 29501366

[B28] Wen L. You C. Lu X. Zhang L. (2015). Phase II trial of concurrent chemoradiotherapy with S-1 versus weekly cisplatin for locoregionally advanced nasopharyngeal carcinoma. Mol. Clin. Oncol. 3 (3), 687–691. 10.3892/mco.2015.529 26137288 PMC4471535

[B29] Wu Q. Zhu C. Zhang S. Zhou Y. Zhong Y. (2021). Hematological toxicities of concurrent chemoradiotherapies in head and neck cancers: comparison among cisplatin, nedaplatin, lobaplatin, and nimotuzumab. Front. Oncol. 11, 762366. 10.3389/fonc.2021.762366 34746003 PMC8566976

[B30] Yau T. K. Lee A. W. Wong D. H. Pang E. S. Ng W. T. Yeung R. M. (2006). Treatment of Stage IV(A-B) nasopharyngeal carcinoma by induction-concurrent chemoradiotherapy and accelerated fractionation: impact of chemotherapy schemes. Int. J. Radiat. Oncol. Biol. Phys. 66 (4), 1004–1010. 10.1016/j.ijrobp.2006.06.016 17145529

[B31] You R. Sun R. Hua Y. J. Li C. F. Li J. B. Zou X. (2017). Cetuximab or nimotuzumab plus intensity-modulated radiotherapy versus cisplatin plus intensity-modulated radiotherapy for stage II-IVb nasopharyngeal carcinoma. Int. J. Cancer 141 (6), 1265–1276. 10.1002/ijc.30819 28577306

[B32] Yu S. Yang Q. Wu J. Zhu M. Ai J. Zhang H. (2021). Clinical application of Epstein-Barr virus DNA loads in Epstein-Barr virus-associated diseases: a cohort study. J. Infect. 82 (1), 105–111. 10.1016/j.jinf.2020.11.027 33248217

[B33] Zhang L. Zhao C. Peng P. J. Lu L. X. Huang P. Y. Han F. (2005). Phase III study comparing standard radiotherapy with or without weekly oxaliplatin in treatment of locoregionally advanced nasopharyngeal carcinoma: preliminary results. J. Clin. Oncol. 23 (33), 8461–8468. 10.1200/jco.2004.00.3863 16230677

[B34] Zheng R. S. Zhang S. W. Zeng H. M. Wang S. M. Sun K. X. Chen R. (2022). Cancer incidence and mortality in China, 2016. J. Natl. Cancer Cent. 2 (1), 1–9. 10.1016/j.jncc.2022.02.002 39035212 PMC11256658

[B35] Zhi-Qiang W. Qi M. Ji-Bin L. Rui Y. You-Ping L. Rui S. (2019). The long-term survival of patients with III-IVb stage nasopharyngeal carcinoma treated with IMRT with or without Nimotuzumab: a propensity score-matched analysis. BMC Cancer 19 (1), 1122. 10.1186/s12885-019-6156-5 31744469 PMC6862826

